# Clinical significance of subepithelial growth patterns in non-muscle invasive bladder cancer

**DOI:** 10.1186/1471-2490-11-17

**Published:** 2011-08-05

**Authors:** Makito Miyake, Shuya Hirao, Hisakazu Mibu, Masahiro Tanaka, Kenji Takashima, Keiji Shimada, Kazuya Hirao

**Affiliations:** 1 Department of Urology, Hirao Hospital, 6-28 Hyobu-cho, Kashihara-shi, Nara, Japan; 2 Department of Urology, Osaka Kaisei Hospital, 1-6-10 Miyahara Yodogawa-ku, Osaka-shi, Osaka, Japan; 3 Takashima Urologic Clinic, 1-1-5 Naizen-cho, Kashihara-shi, Nara, Japan; 4 Department of Pathology, Nara Medical University, 840 Shijyo-cho, Kashihara-shi, Nara, Japan

**Keywords:** bladder cancer, endophytic growth pattern, prognostic factor, infiltrative pattern

## Abstract

**Background:**

We evaluated the clinical significance and prognostic value of histopathological features of bladder cancer, such as subepithelial growth patterns and tumor growth pattern at the invasion front.

**Methods:**

In total, 130 patients newly diagnosed with non-muscle invasive bladder cancer and underwent transurethral resection between 1998 and 2009 were enrolled. Subepithelial growth patterns consisting of endophytic growth pattern (EGP) and von Brunn's nest involvement (VBNI) were investigated using hematoxylin and eosin-stained slides, and their frequency of occurrence, prognostic value, and correlation with other clinicopathological features was evaluated.

**Results:**

EGP and VBNI were found in 40 (30.8%) and 5 (3.9%) of the 130 cases, respectively. Of the 26 pT1 tumors, the growth pattern at the invasion front was trabecular in 17 (65.4%) and infiltrative in 9 (34.6%). Although 8 (47.1%) of 17 trabecular tumors coexisted with EGP, no cases with infiltrative tumors had EGP (p = 0.023). VBNI correlated with high tumor grades (p = 0.006) and lymphovascular involvement (p = 0.026). The multivariate Cox proportional hazards analysis revealed that tumor diameter less than 3 cm (p = 0.04) and intravesical bacillus Calmette-Guérin therapy (p = 0.004) were independent favorable prognostic factors for recurrence-free survival, whereas tumor stage was an independent poor prognostic factor for disease progression (p = 0.006).

**Conclusions:**

Subepithelial growth patterns were not a significant prognostic factor in this study. Additionally, no tumors with an infiltrative growth pattern coexisted with EGP, suggesting that determining the presence of EGP might be helpful for managing non-muscle invasive bladder cancers.

## Background

Urothelial carcinoma (UC) of the bladder is a malignant neoplasm characterized by heterogenous cell populations and divergent clinical outcomes. Approximately 70% of newly diagnosed bladder cancers are non-muscle invasive bladder cancers (NMIBCs) (pTa-1 or pTis), for which the initial treatment is trans-urethral resection of bladder tumor (TURBT). However, 50-70% of these patients experience intravesical recurrence within 5 years, and almost 10% progress to muscle invasive (≥ pT2) or metastatic disease [1]. In the management of NMIBC, standard clinicopathological criteria have been used to assess the risk of intravesical recurrence and disease progression. A recent study by Gofrit et al. demonstrated that subepithelial growth patterns are found predominantly in high-grade and high-stage bladder cancers and are associated with poor prognosis [2]. Subepithelial growth patterns in bladder cancer consist of endophytic growth pattern (EGP) and von Brunn's nest involvement (VBNI).

Although most papillary urothelial carcinomas are characterized by an exophytically growing tumor; some exhibit an EGP and subepithelial growth pattern. A study by Amin et al. reported detailed morphological descriptions of 18 cases of UC with EGP and reviewed the problems associated with assessment of tumor invasion [3]. At present, there is little data on the frequency and prognostic significance of EGP [2,3]. Von Brunn's nests are clusters of urothelial cells within the lamina propria that have become detached from the overlying epithelium [4]. These nests have been commonly identified in 80-90% of normal bladders in autopsy studies [4,5]. A retrospective study demonstrated that VBNI lesions occurred in 73/371 (19.1%) patients with NMIBC, and its presence was not a risk factor for disease progression nor an absolute indication for radical cystectomy [5]. However, since the first report describing VBNI by Seemayer et al. [6], there have been very few reports on its clinical significance [2,5].

In bladder pathology, molecular mimicry between the subepithelial growing tumor and the tumor invading the lamina propria exists. Both of subepithelial growing patterns and appearance of invasion to the lamina propria are morphologically pushing into the stromal tissue. Thus, we aimed to investigate the association between EGP or VBNI and the tumor growth pattern at the invasion front (hereafter referred to as "growth pattern"). According to the classification by Jiminez et al. [7], 3 growth patterns were detected: "nodular" (composed mostly of well delineated, round nests of tumor cells); "trabecular" (composed of broad trabeculae, which usually anastomosed with each other); and "infiltrative" (composed of infiltrating narrow cords or single cells). Growth pattern has been reported to be a poor prognostic factor, both in MIBC and NMIBC [7-10].

In the present study, we retrospectively reviewed the records of 130 patients with newly diagnosed NMIBC in order to elucidate the clinical relevance of subepithelial growth patterns. In pT1 tumors, the association between tumor growth pattern at the invasion front and subepithelial growth pattern was also analyzed.

## Methods

### Patients and pathological review

An institutional database was obtained from the Hirao Hospital registry, and archived data on patients newly diagnosed with NMIBC between July 1998 and November 2009 were reviewed. All of the patients were ethnically Japanese. Patients with concomitant urothelial carcinoma of the upper urinary tract or nonurothelial carcinoma histology were excluded. All hematoxylin and eosin-stained slides were reviewed by a single uropathologist (K.S.) for staging (according to the TNM system) [11]; grading (according to the 2004 WHO classification) [12]; presence of EGP, VBNI, carcinoma *in situ *(CIS) and lymphovascular involvement (LVI); and determination of the growth pattern in the 26 cT1 tumors. Tumors were classified into papillary urothelial neoplasm with low malignant potential (PUNLMP), low grade (LG), and high grade (HG). EGP was defined as the presence of either broad bulbous tongues of neoplastic urothelial cells in the presence or absence of stromal reaction extending deep into the lamina propria but not into the muscularis propria or endophytic expansile growth with or without peripheral palisading of basal cells [3]. The use of clinicopathological information was approved by the Local Commission for Medical Ethics and Clinical Studies.

### Follow-up after initial TURBT

Clinical information and follow-up data were collected by chart review. Adjuvant intravesical instillation therapy after the initial TURBT was administered to 75 patients. Of these, 67 received intravesical instillation of bacillus Calmette-Guérin (BCG) 6 or 8 times at a dose of 80 mg. The remaining 8 patients received intravesical instillation of an anthracycline antitumor drug, epirubicin, 30 times at a dose of 20 or 40 mg. Follow-up management was relatively uniform. The patients were monitored by routine cystoscopy and urine cytology at 3-month intervals during the first 3 years, every 6 months during the following 2 years, and thereafter once every year. Abdominopelvic computerized tomography and chest radiography were performed at 6- to 12-month intervals. Recurrence was defined as the histological diagnosis of a tumor in the urinary bladder after surgical resection. Progression was defined as the detection of a muscle-invasive disease (≥ pT2) in the urinary bladder or a metastatic site in other organs by histological diagnosis and/or diagnostic imaging.

### Statistical analysis

Dr. SPSS II version 11.0.1 for Windows (SPSS Inc., Chicago, IL, USA) and PRISM software version 4.00 (San Diego, CA, USA) were utilized for statistical analyses and plotting the data, respectively. A p value of < 0.05 was considered statistically significant. Correlation among the clinicopathological variables was analyzed using the chi-square test of association. Recurrence-free and progression-free survival curves were obtained using the Kaplan-Meier method, and compared by the log rank test for each prognostic variable. Multivariate analysis was performed to identify independent prognostic variables using a stepwise Cox proportional hazards regression model. For multivariate analysis, variables were selected on the condition that they were statistically significant in the univariate analysis.

## Results

### Correlation between subepithelial growth patterns and other clinicopathological parameters

**Figure 1:**
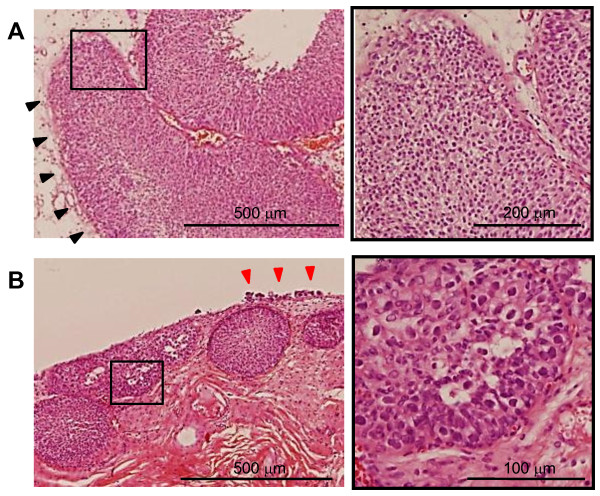
**Representative pathological findings with subepithelial growth patterns**. (A) An endophytic growth pattern (black arrowheads) is observed with a low-grade papillary growing tumor. (B) A high-grade tumor with Brunn's nests. Carcinoma *in situ *is seen on the surface epithelium (red arrowheads). The right panel represents a magnification of the box marked in the left panel.

EGP and VBNI were found in 40/130 (30.8%) and 5/130 (3.9%) patients, respectively (Figure 1). We determined the association of EGP and VBNI with various clinicopathological parameters (Table 1). VBNI correlated with high tumor grade (p = 0.006) and the presence of LVI (p = 0.026), whereas EGP did not correlate with any of the parameters analyzed. The growth pattern was trabecular in 17/26 (65.4%) and infiltrative in 9/26 (34.6%) pT1 tumor patients. It is significant that no tumor with an infiltrative growth pattern coexisted with EGP. In contrast, 8/17 (47.1%) tumors with a trabecular growth pattern were found to coexist with EGP (p = 0.023).

**Table 1:** The association between subepithelial growth patterns and various parameters

Variables		Cases (n)	Subepithelial growth pattern							
			
			Endophytic growth pattern				von Brunn's nests involvement			
				
			Absent	Present	Present (%)	p value	Absent	Present	Present (%)	p value
										
**Total**		130	90	40	30.8	-	125	5	4.0	-
										
**Age (years)**						0.56				0.66
	< 70	55	35	20	36.4		52	3	5.5	
	≥70	65	45	20	30.8		63	2	3.1	
										
**Gender**						0.96				1.00
	M	114	79	35	30.7		109	5	4.4	
	F	16	11	5	31.3		16	0	0.0	
										
**T stage**						1.00				0.054
	a	104	72	32	30.8		102	2	1.9	
	1	26	18	8	30.8		23	3	11.5	
										
**Tumor grade (2004 WHO)**						0.70				0.006
	PUNLMP	13	10	3	23.1		13	0	0.0	
	LG	84	55	29	34.5		83	1	1.2	
	HG	33	25	8	24.2		25	4	12.1	
										
**Concomitant CIS**						0.054				0.26
	absent	123	73	40	35.4		109	4	3.5	
	present	7	7	0	0.0		6	1	14.3	
										
**Lymphovascular involvement**						0.94				0.026
	absent	110	76	34	30.9		108	2	1.8	
	present	20	14	6	30.0		17	3	15.0	
										
**Tumor growth pattern**						0.023				0.53
**(pT1, n = 26)**	trabecular	17	9	8	47.1		14	3	17.6	
	infiltrative	9	9	0	0.0		9	0	0.0	
										
**Endophytic growth pattern**										0.64
	absent	90	-	-	-		87	3	3.3	
	present	40	-	-	-		38	2	5.0	
										
**von Brunn's nest involvement**						0.64				
	absent	125	87	38	30.4		-	-	-	
	present	5	3	2	40.0		-	-	-	
										
**Multiplicity**						0.33				0.66
	solitary	70	51	19	27.1		68	2	2.9	
	multiple	60	39	21	35.0		57	3	5.0	
										
**Tumor diameter (cm)**						0.17				0.59
	< 3	99	80	28	28.3		96	3	3.0	
	3 ≤	31	19	12	38.7		29	2	6.5	

### Analyses of prognostic parameters for intravesical tumor recurrence and disease progression

To examine the relevance of subepithelial growth patterns in disease outcome, we performed univariate and multivariate analyses using prognostic parameters. The median follow-up period was 36 months (range: 1 to 140 months). In all, 41/130 (31.5%) and 6/130 (4.6%) patients experienced recurrence and progression, respectively. The univariate analysis for tumor recurrence revealed that multiplicity (p = 0.038) and tumor diameter ≥ 3 cm (p = 0.023) were significant predictors of a high recurrence rate, and that intravesical BCG therapy (p = 0.039) decreased the recurrence rate (Table 2). The multivariate Cox proportional hazards analysis revealed that both, tumor diameter < 3 cm (p = 0.04) and intravesical BCG therapy (p = 0.004) were independent favorable prognosis parameters of recurrence-free survival (Table 2). Upon evaluating factors associated with disease progression, the univariate analysis demonstrated that stage (p < 0.0001), tumor grade (p = 0.0013), LVI (p = 0.0002), and tumor diameter ≥ 3 cm (p = 0.0004) were significantly associated with a poor prognosis (Table 3). Among these, stage was the only independent factor associated with disease progression (p = 0.006, Table 3). However, the number of disease progression events was too small for multivariate analysis.

**Table 2:** Univariate and multivariate analysis of intravesical tumor recurrence

Variables		Cases (n)	Tumor recurrence			
			
			Univariate		Multivariate	
				
			Hazard ratio (95% CI)	p value	Hazard ratio (95% CI)	p value
**T stage**						
	a	104	1	-		
	1	26	1.40 (0.68-3.21)	0.32		
						
**Tumor grade (2004 WHO)**				0.14		
	PUNLMP	13	1	-		
	LG	84	1.41 (0.50-4.00)	0.57		
	HG	33	2.17 (0.70-5.56)	0.2		
						
**Concomitant CIS**						
	absent	123	1	-		
	present	7	1.32 (0.36-5.28)	0.20		
						
**Lymphovascular involvement**						
	absent	110	1	-		
	present	20	1.75 (0.86-4.93)	0.11		
						
**Endophytic growth pattern**						
	absent	90	1	-		
	present	40	0.76 (0.39-1.52)	0.45		
						
**von Brunn's nest involvement**						
	absent	125	1	-		
	present	5	1.54 (0.29-9.94)	0.54		
						
**Multiplicity**						
	solitary	70	1	-	1	-
	multiple	60	1.91 (1.04-3.59)	0.038	1.93 (0.98-3.79)	0.058
						
**Tumor diameter (cm)**						
	< 3	99	1	-	1	-
	≥ 3	31	2.04 (1.13-5.06)	0.023	2.10 (1.04-4.27)	0.040
						
**Intravesical therapy**						
	None	55	1	-	1	-
	BCG	67	0.53 (0.23-0.96)	0.039	0.35 (0.18-0.71)	0.004
	Anthracyclines	8	0.96 (0.28-3.21)	0.94	0.63 (0.24-2.38)	0.63

**Table 3:** Univariate and multivariate analysis for disease progression

Variables		Cases (n)	Disease progression			
			
			Univariate		Multivariate	
				
			Hazard ratio (95% CI)	p value	Hazard ratio (95% CI)	p value
**T stage**						
	a	104	1	-	1	-
	1	26	20.69 (7.82-446.23)	< 0.0001	20.94 (2.44-179.45)	0.006
						
**Tumor grade (2004 WHO)**						
	PUNLMP/LG	97	1	-	1	-
	HG	33	14.37 (3.25-126.40)	0.0013	2.97 (0.098-90.16)	0.53
						
**Concomitant CIS**						
	absent	123	1	-		
	present	7	ND	0.51		
						
**Lymphovascular involvement**						
	absent	110	1	-	1	-
	present	20	12.07 (7.49-733.68)	0.0002	1.23 (0.13-11.28)	0.86
						
**Multiplicity**						
	solitary	70	1	-		
	multiple	60	5.44 (0.84-20.60)	0.082		
						
**Tumor diameter (cm)**						
	< 3	99	1	-	1	-
	≥ 3	31	16.93 (4.63-209.78)	0.0004	6.77 (0.69-66.8)	0.10
						
**Endophytic growth pattern**						
	absent	90	1	-		
	present	40	ND	0.096		
						
**von Brunn's nest involvement**						
	absent	125	1	-		
	present	5	5.82 (0.74-5617.98)	0.068		
						
**Intravesical therapy**						
	None	55	1	-		
	BCG	75	1.09 (0.20-5.99)	0.92		

**Figure 2:**
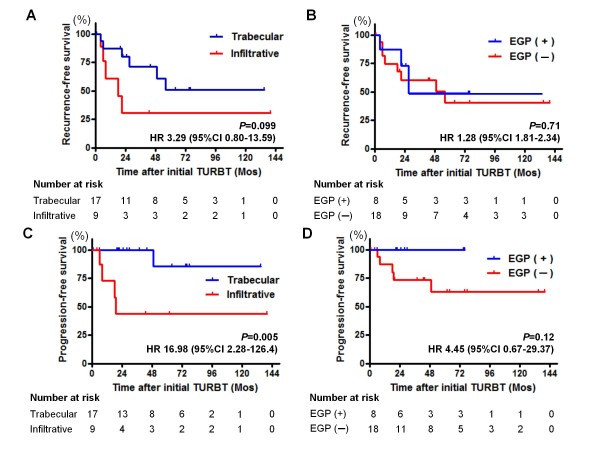
**Kaplan-Meier curves for intravesical recurrence (A, B) and disease progression (C, D) of patients with pT1 tumors (n = 26)**. A comparison of tumor growth patterns at the tumor invasion front, trabecular vs. infiltrative and presence vs. absence of endophytic growth pattern (EGP) is displayed. The numbers at risk are shown below each graph.

In the 26 pT1 tumors, EGP was associated with the growth pattern typical of less aggressive tumors (Table 1), suggesting that EGP may be a favorable prognostic factor in patients with a pT1 tumor. We performed prognostic analyses in a subset of pT1 tumors using the Kaplan-Meier method and log-rank test. Neither the growth pattern nor EGP was associated with recurrence-free survival rate (Figure 2A and 2B). In the absence of EGP, there was a tendency toward disease progression, and an infiltrative growth pattern was associated with a high risk of progression (Figure 2C and 2D). Growth patterns were the only significant predictors of progression.

## Discussion

In the management of NMIBC, standard clinicopathological criteria such as multiplicity, tumor diameter, prior recurrence rate, T stage, tumor grade, and presence of concomitant CIS have been used to assess the risk of intravesical recurrence and progression to MIBC [13,14]. Histopathological features of UC including subepithelial growth patterns and tumor growth pattern at the invasion front are additional prognostic factors. In the present study, we have investigated the frequency and prognostic relevance of subepithelial growth patterns and tumor growth patterns in 130 cases with NMIBC. To our knowledge, this is the first study to analyze these histopathological variants.

A higher rate of EGP has been reported in high-grade tumors as compared to low-grade tumors [2]. In our cohort, there was no significant correlation between EGP and tumor grade. Moreover, we found a higher incidence of EGP as compared to that reported by Gofrit et al. (30.8% vs. 11.9%). Several factors can account for this discrepancy. Artifact from difference in pathologic interpretation cannot be ruled out and further analysis on confirmatory review by a secondary pathologist would be required. In addition, various environmental factors are associated with carcinogenesis of bladder cancer and the degree of exposure to these environmental carcinogens may vary in different countries and cultures [15]. Moreover, susceptibility to carcinogens also depends on genetic background and such differences could contribute to carcinogenesis of bladder cancer and the subsequent discrepancy in the incidence of EGP. Additionally, in contrast to the previous reports [2], EGP was not identified as a significant indicator of tumor recurrence or progression. However, it is interesting to note that EGP was associated with the tumor growth pattern at the invasion front in pT1 tumors (Table 2). Jimenez et al. were the first to report that the infiltrative growth pattern of MIBC was associated with poor survival [7]. Recently, Denzinger et al. reported that the growth pattern in a series of pT1G3 tumors was a strong predictor of cancer-specific survival and could thus be important in planning therapeutic strategies [10]. Similarly, our data revealed that the infiltrative growth pattern was related to worse progression-free survival as compared to the trabecular growth pattern (Figure 2C). Although there was no statistical significance in the recurrence- and progression-free survival between tumors with and without EGP, it was a notable finding that none of the tumors with EGP underwent disease progression (Figure 2D). Further studies are warranted to clarify the potential prognostic power of EGP in pT1 tumors.

The infiltrative growth pattern is characterized by single cell infiltration of the invasive front of tumors, possibly due to a loss of cell cohesion. In other malignant diseases, a loss of adhesional markers was seen in the epithelial mesenchymal transition (EMT) phenomenon, associated with poor prognosis [16]. Initial studies on EMT in bladder cancer suggested that low levels of the adhesion molecules plakoglobin and β-catenin were related to worse prognosis [17]. Thus, we suggest that tumors with EGP may have a tendency to retain the expression and function of the adhesion molecules.

A high prevalence of von Brunn's nests (89%) was shown in the normal urothelium. However, VBNI was present in only 3.9% of our subjects: in 1/83 (1.2%) LG tumors, 4/25 (12.1%) HG tumors, and none of the 13 PUNLMP cases (p = 0.006, Table 1). VBNI was significantly correlated with high tumor grade. There are conflicting reports on the prognostic value of VBNI in disease progression [2,5]. We could not confirm any of the previous findings due to the small number of subjects with VBNI. In our study, of the 5 cases with VBNI, 1 received no adjuvant treatment and experienced intravesical tumor recurrence a year after the initial TURBT. Of the 4 patients that received intravesical BCG treatment, 3 showed neither recurrence nor progression during the follow-up duration. However, 1 patient experienced disease progression, wherein the tumor extended into the prostatic duct 4 years after the initial TURBT. For this case, an early radical cystectomy would be the optimal treatment. However, the clinical relevance of VBNI as a prognostic marker and therapeutic guide remains unclear.

There are several limitations in our study. Our results were based on the retrospective analysis from a small number of patients. In particular, the numbers of patients who experienced disease progression, total cystectomy or cancer-specific death were too low to generate an accurate analysis. The prognostic significance of subepithelial growth patterns were not compared to cancer-specific survival and overall survival in our study. Additionally, since the recognition degree of EGP is considered low by both urologists and pathologists of our country, the difference in pathologic interpretation could affect our results. Therefore, confirmatory pathologic reviews should be established to consolidate the entity and clinical significance of EGP.

## Conclusions

Subepithelial growth patterns were not a significant prognostic factor in this study. Considering that no tumors with an infiltrative growth pattern coexisted with EGP, determining the presence of EGP might be helpful for managing NMIBC. Our report would provide an opportunity for researchers to promote the further evaluation of EGP and VBNI. Therefore, large-scale studies and prospective clinical trials are necessary to confirm the clinical significance of these pathologic features. We believe that validation studies on subepithelial growth patterns will aid in the prognosis and treatment of urothelial cell cancers.

## Competing interests

The authors declare that they have no competing interests.

## Authors' contributions

MM was involved in conceiving the study, carried out acquisition of data and drafted the manuscript. HS and MH performed the statistical analysis and interpretation of data. SK conceived the study and supervised histopathological analysis of tumour specimens. TM, TK and HK have been involved in drafting and revising the manuscript critically. All authors read and approved the final manuscript.

## Pre-publication history

The pre-publication history for this paper can be accessed here:

http://www.biomedcentral.com/1471-2490/11/17/prepub
